# Sexual Dimorphism in Fin Size and Shape in Bluefin Killifish

**DOI:** 10.1002/ece3.73036

**Published:** 2026-02-12

**Authors:** Kasey Brockelsby, Elijah J. Davis, Olivia A. Roden, Valerie Shamsyna, Rebecca C. Fuller

**Affiliations:** ^1^ Department of Evolution, Ecology, and Behavior, School of Integrative Biology University of Illinois Urbana Illinois USA; ^2^ School of Integrative Biology University of Illinois Urbana Illinois USA

**Keywords:** coefficient of variation, fin rays, fins, morphological correlation, sexual dimorphism, sexual selection

## Abstract

Sexual dimorphism provides insight into how trait optima differ between males and females, despite their shared genome. Measuring sexual dimorphism can help identify which traits have been shaped by sexual selection. While fish morphology has been widely described, fewer studies have quantified sexual dimorphism across all fin types—pectoral, pelvic, dorsal, anal, and caudal. Fins are often overlooked due to their small size, tendency to fold against the body, and poor preservation post‐collection. In this study, we quantified sexual dimorphism in fin size and shape across all fin types in the bluefin killifish, *Lucania goodei* . We found striking sexual dimorphism in the dorsal and anal fins, particularly in area, ray length, and base length. In contrast, the pelvic, pectoral, and caudal fins showed moderate but detectable levels of dimorphism. In both dorsal and anal fins, males exhibited elongation of the posterior region relative to females. Dorsal and anal fin traits (i.e., area, ray length, base length) were also strongly correlated within both sexes, such that individuals with larger than average dorsal fins also have larger than average anal fins. This correlation is present for both males and females, suggesting shared developmental pathways, pleiotropy, or correlated selection between dorsal and anal fins. Overall, our results indicate that the dorsal and anal fins are key targets of sexual selection in males, likely reflecting their roles in courtship, competition, and/or external fertilization.

## Graphical abstract

This study shows very high levels of sexual dimorphism in dorsal and anal fin traits compared to other fins. Dorsal and anal fin traits are also correlated, such that individuals with large dorsal fins also have large anal fins. Together, this suggests strong sexual selection on these functionally integrated traits.

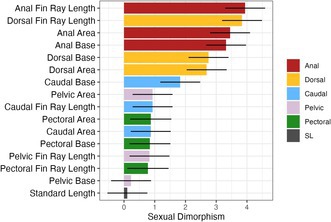

## 1 Introduction

Sexual dimorphism is common across fishes (Breder and Rosen 1966; Andersson 1994). Males and females often differ in coloration, presence/absence of breeding tubercles, body size/shape, vocalizations, and behavior. Fewer studies have focused on sexual dimorphism in fin size and shape, although notable exceptions exist in some groups (Im et al. 2016; Goldberg et al. 2019; Sowersby et al. 2022). Fins are intriguing because they are multifunctional. They affect multiple aspects of swimming, including stabilization in the water column, forward thrust, and turning (Standen and Lauder 2005; Flammang and Lauder 2016). Fins are also utilized in mating, including the courtship of females, displays towards competing males, and the movement and coordination of mating during pair spawning (Breder and Rosen 1966; Basolo 1990; Rosenthal and Evans 1998; Kozak et al. 2008). Some species even use their fins to “clasp” females during spawning (Able and Hata 1984; Sabaj et al. 2000; Hayakawa and Kobayashi 2010). Measuring sexual dimorphism is valuable because it can indicate which traits are under sexual selection (Kodric‐Brown 1985; Williams et al. 2013; Delhey et al. 2023) and provide insight into the specific functions of the various fins.

A sizable literature examines the evolution of fish body morphology, fin placement, and length of spiny rays as a function of ecology (Langerhans 2008; Chang and Alfaro 2016; Price et al. 2019). However, less attention has been given to the fins, particularly to the soft ray fins (Standen and Lauder 2005). Soft‐rayed fins often lie flat against the body when fish are removed from water. Geometric morphometrics techniques are more challenging to apply to soft ray fins because they have fewer non‐flexible landmarks, making size and shape difficult to quantify. One solution is to measure the lengths of multiple fin rays (or all) that span the fin along with the length of the fin base (Davis et al. 2025). This allows for standardized insights into fin size and shape.

Despite these challenges, sexual dimorphism in fin size and shape is documented, including the extreme dimorphisms among livebearing fishes, with the enlarged dorsal fin of sailfin mollies ( *Poecilia reticulata* ) and the elongated caudal fin found in sword tail fishes (*Xiphophorus*) (Basolo 1990; Rosenthal and Evans 1998; Rosenthal et al. 2001; Kozak et al. 2008). Other members of the Poeciliidae are also known for having dimorphic dorsal fins (Goldberg et al. 2019). However, sexual dimorphism in fin traits may be common across the order Cyprinodontiformes, to which poeciliids belong. Recently, Davis et al. (2025) measured sexual dimorphism across 20 species of North American killifish (Fundulidae) and found pronounced dimorphism in dorsal and anal fin traits, but modest dimorphism in caudal fin traits (see also Welsh et al. 2013; Welsh and Fuller 2015). Pronounced sexual dimorphism in dorsal and anal fin traits has also been found in other families of killifish (Sowersby et al. 2022; Mainero et al. 2023). Mainero et al. (2023) examined the length of fins in the Arabian killifish, *Aphaniops stoliczkanus*, and found sexual dimorphism in anal, dorsal, pectoral, and pelvic fin lengths and base lengths when scaled to standard length. Anal and dorsal fins had particularly high levels of sexual dimorphism in comparison to the other fins. A closely related order (Beloniformes) contains the medaka, which also possesses pronounced dimorphism in dorsal and anal fins in some populations (Sumarto et al. 2020; Downer‐Bartholomew and Rodd 2025). Across teleosts, many fish taxa have sexually dimorphic fins with different fins possessing high levels of sexual dimorphism in different groups (Echeverria 1986; Brichard 1989; Bronseth and Folstad 1997; Karino 1997; Bakker and Mundwiler 1999; Casselman and Schulte‐Hostedde 2004; Skjæraasen et al. 2006). These patterns have been attributed to the various demands of attracting mates, competing with males, fertilizing eggs, and caring for offspring as well as differences in microhabitats between males and females.

Fins provide an excellent tapestry for investigating classic questions regarding natural and sexual selection and the nature of genetic constraints. In addition to being multifunctional traits, they also provide a compelling system to pose basic questions about trait correlations and trait variability (Falconer 1996). Basic considerations of allometry predict that traits scale positively with size. High levels of pleiotropy and/or shared developmental pathways between fins predict high correlations between traits, even after correcting for overall size. The extent to which trait variability itself differs between the sexes or fin types is also relevant to our understanding of selection. Some theories predict that condition‐dependent traits, which are often exaggerated in males, should exhibit high variability (Rowe and Houle 1996; Houle and Kondrashov 2002; Zajitschek et al. 2020), which have been found in some fish (Orbach et al. 2019; Mainero et al. 2023). Conversely, traits under strong directional or stabilizing selection are expected to have low levels of genetic variation. By comparing variability across traits and between sexes, we can assess whether patterns of variation align with predictions based on condition‐dependent sexual signaling and general selective pressures (Kodric‐Brown and Hohmann 1990).

This study sought to estimate the levels of sexual dimorphism across all fin types (dorsal, anal, caudal, pectoral, and pelvic) and to determine the relative levels of variability and covariation. To answer these questions, we focused on the bluefin killifish, *Lucania goodei* , which are known for displaying high levels of sexual dichromatism in their fins; males have brightly colored dorsal and anal fins (Foster 1967; Fuller 2002; Fuller et al. 2022) that are signals of condition and dominance (Johnson and Fuller 2015). The pelvic fins and the caudal fin base are also brightly colored in males. By contrast, female bluefin killifish are drab, with minimal fin color. The pectoral fins of both species exhibit no color at all in either sex.

In this study, we asked the following: Which fins show the highest levels of sexual dimorphism in size and/or shape? One possibility is that generalized sexual selection would lead to larger fins in males, such that fins with nuptial coloration (dorsal, anal, pelvic, and caudal) would have higher levels of dimorphism in morphology. Alternatively, fins that are involved in clasping during spawning may evolve to be enlarged in males. Do males have higher levels of trait variability than females in fins with high levels of dimorphism? If fin size serves as a condition‐dependent signal, then traits with high sexual dimorphism might also exhibit high variability. Are there tight correlations between trait values that suggest pleiotropy and/or a shared developmental pathway?

To answer these questions, we took high‐resolution pictures of male and female bluefin killifish ( *Lucania goodei* ) and measured the area and base length of the fin as well as all of the fin ray lengths of the dorsal, anal, caudal, pelvic, and pectoral fins. Measuring the base lengths and fin ray lengths allowed us to determine which components contributed to differences in fin area and fin shape. We found that dorsal and anal fins are particularly dimorphic and covary with one another, suggesting that males with large dorsal fins also have large anal fins, even after controlling for overall size. We also found high correlations between dorsal and anal fin traits in both males and females, suggesting the presence of pleiotropy, shared developmental pathways, and/or correlated selection.

## 2 Methods

Our goal was to measure sexual dimorphism in fin size and shape in the bluefin killifish, *Lucania goodei*, to determine whether sexually dimorphic traits had higher (or lower) levels of variability, and to determine whether there were strong correlations between different fin elements. Fish were collected from the wild from two different populations in Florida: Rainbow River (clear, spring‐fed river, Marion County) and Everglades 26 Mile Bend (swamp, Broward County) populations, in May 2021. The fish were returned to the laboratory and maintained in a greenhouse until Spring 2022. We measured fin size, shape, and standard length for approximately 10 males and 10 females from each population. Individuals were sexed based on diagnostic sexually dimorphic characteristics, namely the presence of color and black melanin spots and/or borders on the dorsal and anal fins of males. Fish were euthanized in a buffered solution of MS‐222 (tricaine mesylate) so that their fins could be manipulated more easily and then photographed.

Fish were photographed in a petri dish with a small amount of water on a plain gray stage, using a Nikon D5600 camera with an AF‐S Micro Nikkor 105 mm lens. To minimize glare and ensure overall quality, two lamps were angled to illuminate the stage with diffuse light. Finally, a small ruler was placed on the stage underneath the petri dish to provide a scale, and male fish were also photographed with a color standard in the frame. At least four photographs were taken of every fish: one photo capturing the full body of the fish, then one photo focused on each unpaired fin (dorsal, anal, and caudal). Once these photos had been taken, the pectoral and pelvic fins of the fish were cut off with small dissecting scissors and placed on a microscope slide for further photography. Microscope slides were placed on the gray stage under the same lighting conditions with a small ruler for scale. We took photos focused on the pelvic and pectoral fins.

**FIGURE 1:**
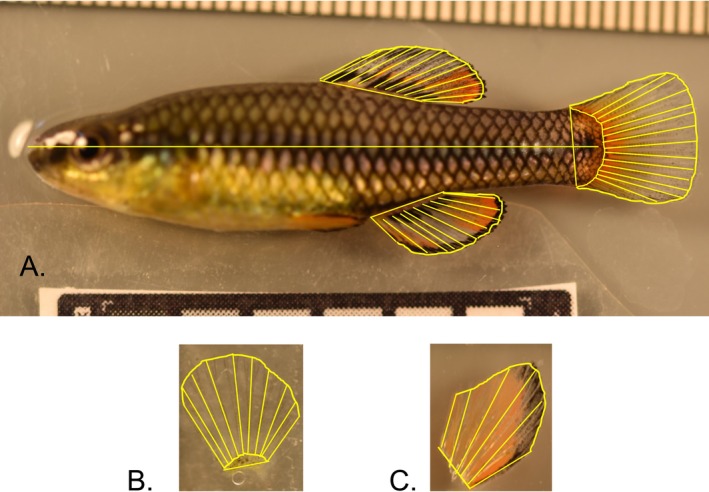
(A) Male *L. goodei* with measurements shown for standard length and dorsal, anal, and caudal fins traits (fin base length, area, ray number, and ray length); (B, C) pelvic and pectoral fin traits (base length, area, fin ray number, fin ray lengths). Photo credit Kasey Brockelsby.

Measurements were obtained using ImageJ ver. 1.53 k. We measured standard length (tip of the snout to the base of the caudal fin), length of the base of each fin (insertion of the first ray to the insertion of the last ray), lengths of individual fin rays within each fin, and area of each fin. The number of rays in each fin was also counted. Figure 1 shows an example of linear measurements taken on a specimen. Caudal fin ray counts and lengths were measured following the protocols described by Armbruster (2012). Specifically, we measured the median caudal ray and all branched caudal rays plus one unbranched ray adjacent to the branched rays on both the dorsal and ventral sides. These measurements were performed twice by separate people. We measured fin area, fin base length, number of fin rays, and fin ray lengths for pectoral and pelvic fins using the images of fins removed from the body.

### 2.1 Data Analysis

We calculated the repeatability of measurements taken by multiple investigators and multiple photos of the same specimen. Dorsal and anal fin traits were measured by two separate people using two separate photos (i.e., four total measurements per trait per individual). All other traits were measured using a single photo measured by two people (i.e., two total measurements per trait per individual). Repeatability was calculated using the “lmer” function from the “lmer4” library in R, where we treated each individual fish as a random factor.

The overarching goals of this project were to determine (1) which fins (i.e., dorsal, anal, caudal, pectoral, or pelvic) and fin traits (i.e., base length, ray length, area) show the highest levels of sexual dimorphism, (2) whether some fins are particularly variable and whether this varies as a function of sex, and (3) whether there are strong correlations across these traits independent of size. We considered individuals as the unit of replication. Multiple measurements of the same trait on the same individual were averaged to produce a single value. For each fin, we also calculated the mean ray length for all rays. Fin area, fin ray lengths, and base lengths were all positively correlated with standard length. To examine fin size and shape independent of overall size, we calculated the residuals from linear regressions of fin areas, base lengths, and ray lengths against mean standard length. We then used a linear model to test for differences in residual trait variation due to sex, source population, and an interaction between the two. Only one trait varied significantly as a function of the interaction between sex and population, and this test did not remain statistically significant after a sequential Bonferroni correction. Hence, we ran a simple additive model that considered the effects of sex and population, but not their interactions.

Sexual dimorphism was measured as the effect size due to sex. The effect was measured with the “eff_size” function from the “emmeans” library in R. This is the difference in means between the two sexes divided by the pooled standard deviation. We also calculated the 95% confidence limits to ask whether sexual dimorphism differed from zero and whether it differed from other traits.

To visualize sex differences in fin size and shape, we calculated the least‐square means and standard errors for each ray length and the base length for all the fins from separate models that considered the effects of standard length and sex on trait value. To visualize the fins, the first fin ray was located at the origin (0), the last ray was located at the end of the sex‐specific fin base, and the intermediate rays were evenly spaced between the first and last rays. This allowed us to visualize sex differences in base length, fin ray lengths, and shape.

To determine whether the two sexes differed in the levels of variability for a given trait, we estimated the sex‐specific absolute means and coefficients of variation on the raw data as well as the size‐corrected coefficients of variation. For the size‐corrected coefficients of variation, we calculated the standard deviation of the residuals from a linear regression of trait value on standard length for both males and females and then divided the standard deviations by the absolute means. We implemented the LeveneTest to test for differences in variability in the raw values. We also saved the residuals from a regression of the trait on standard length and used the LeveneTest to compare variation levels between the sexes. To correct for multiple testing, we implemented a sequential Bonferroni correction.

We also investigated the correlations among size‐corrected fin traits to determine whether there were particularly strong correlations among some fins and whether this pattern differed between males and females. To do this, we calculated the residuals from separate regressions of standard length on trait values for each sex and each trait. We then examined the correlations among fin area, fin ray lengths, and fin base lengths separately for each sex. For each fin trait (i.e., area, ray length, base length) and each sex, we performed a sequential Bonferroni correction.

All analyses were performed in R version 4.5.0. Raw data and R scripts can be found at https://doi.org/10.5061/dryad.80gb5mm2x.

## 3 Results

**FIGURE 2:**
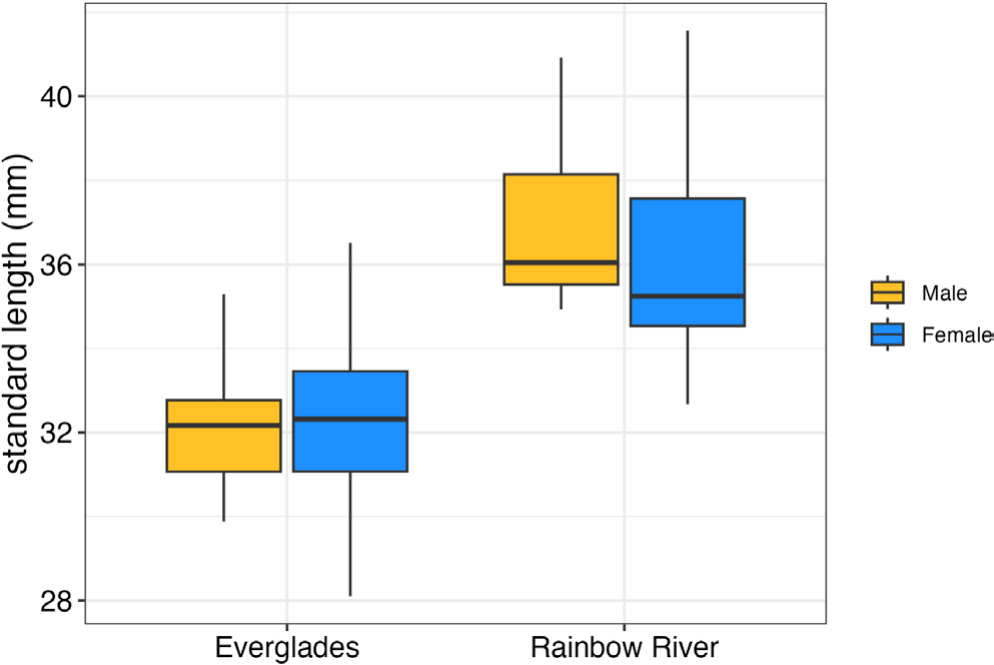
Standard length as a function of sex and population.

Repeatability was generally high (average repeatability = 0.80, Table 1). Most traits were positively correlated with standard length, with an average correlation of 0.54 between standard length and continuous fin traits (i.e., fin area, fin ray lengths, and fin base length, Table 2). Ray counts were not positively correlated with standard length (*p* > 0.27), with the exception of dorsal ray counts (*R*_37_ = 0.33, *p* = 0.034). Analyses of variance indicated that ray counts did not vary as a function of sex or population (*p* > 0.09 in all tests), regardless of whether standard length was taken into account. Finally, standard length varied as a function of population of origin (Figure 2, *F*_1,35_ = 34.08, *p* = 1.263e‐06), but there were no statistically significant effects of sex (*F*_1,35_ = 0.13, *p* = 0.7236) or the interaction between sex and source population (*F*_1,35_ = 0.31, *p* = 0.5830). For the remaining analyses, we consider size‐adjusted trait values as the residual values of traits regressed on standard length.

**TABLE 1:** Repeatability across traits. Repeatability is listed for traits where the sample size across individuals was > 20.

Traits	Repeatability	Total individuals	Total images
Standard length	0.96	39	78
Dorsal Rays #	0.84	39	154
Dorsal Area	0.97	39	154
Dorsal Base Length	0.74	39	154
Dorsal 1 Ray Length	0.80	39	154
Dorsal 2 Ray Length	0.77	39	154
Dorsal 3 Ray Length	0.68	39	154
Dorsal 4 Ray Length	0.86	39	154
Dorsal 5 Ray Length	0.90	39	154
Dorsal 6 Ray Length	0.89	39	154
Dorsal 7 Ray Length	0.93	39	154
Dorsal 8 Ray Length	0.93	39	154
Dorsal 9 Ray Length	0.94	39	154
Dorsal 10 Ray Length	0.94	38	145
Dorsal 11 Ray Length	0.95	27	99
Anal Ray #	0.78	39	153
Anal Area	0.95	39	153
Anal Base Length	0.94	39	153
Anal 1 Ray Length	0.70	39	152
Anal 2 Ray Length	0.61	39	152
Anal 3 Ray Length	0.66	39	152
Anal 4 Ray Length	0.78	39	152
Anal 5 Ray Length	0.82	39	152
Anal 6 Ray Length	0.87	39	152
Anal 7 Ray Length	0.89	39	152
Anal 8 Ray Length	0.90	39	151
Anal 9 Ray Length	0.88	39	151
Anal 10 Ray Length	0.94	34	123
Caudal Ray #	0.89	39	79
Caudal Area	0.96	39	146
Caudal Base Length	0.88	39	144
Median Caudal Ray Length	0.94	39	79
Caudal Dorsal 1 Ray Length	0.81	39	79
Caudal Dorsal 2 Ray Length	0.90	39	79
Caudal Dorsal 3 Ray Length	0.89	39	79
Caudal Dorsal 4 Ray Length	0.92	39	79
Caudal Dorsal 5 Ray Length	0.86	39	79
Caudal Dorsal 6 Ray Length	0.73	39	78
Caudal Dorsal 7 Ray Length	0.65	22	45
Caudal Ventral 1 Ray Length	0.73	39	79
Caudal Ventral 2 Ray Length	0.90	39	79
Caudal Ventral 3 Ray Length	0.90	39	79
Caudal Ventral 4 Ray Length	0.89	39	79
Caudal Ventral 5 Ray Length	0.86	39	79
Caudal Ventral 6 Ray Length	0.75	39	78
Pelvic Rays #	0.61	38	75
Pelvic Area	0.95	39	78
Pelvic Base Length	0.57	39	78
Pelvic 1 Ray Length	0.75	39	78
Pelvic 2 Ray Length	0.72	39	78
Pelvic 3 Ray Length	0.82	39	78
Pelvic 4 Ray Length	0.89	39	78
Pelvic 5 Ray Length	0.78	39	77
Pelvic 6 Ray Length	0.81	34	62
Pectoral Rays #	0.63	37	73
Pectoral Area	0.94	37	73
Pectoral Base Length	0.77	37	73
Pectoral 1 Ray Length	0.55	37	73
Pectoral 2 Ray Length	0.46	37	73
Pectoral 3 Ray Length	0.53	37	73
Pectoral 4 Ray Length	0.61	37	73
Pectoral 5 Ray Length	0.71	37	73
Pectoral 6 Ray Length	0.77	37	73
Pectoral 7 Ray Length	0.70	36	72
Pectoral 8 Ray Length	0.66	35	70
Pectoral 9 Ray Length	0.66	33	64
Pectoral 10 Ray Length	0.45	24	43

**TABLE 2:** Correlation coefficients between standard length and fin traits.

Trait	*R*	DF	*p*
Dorsal Ray #	0.33	37	0.0395
Dorsal Area	0.43	37	0.0065
Dorsal Base Length	0.59	37	7.62E‐05
Mean Dorsal Fin Ray Length	0.51	37	0.0008
Dorsal Ray 1 Length	0.31	37	0.0566
Dorsal Ray 2 Length	0.51	37	0.0010
Dorsal Ray 3 Length	0.59	37	6.80E‐05
Dorsal Ray 4 Length	0.57	37	0.0002
Dorsal Ray 5 Length	0.59	37	7.96E‐05
Dorsal Ray 6 Length	0.54	37	0.0003
Dorsal Ray 7 Length	0.50	37	0.0012
Dorsal Ray 8 Length	0.46	37	0.0030
Dorsal Ray 9 Length	0.50	37	0.0011
Dorsal Ray 10 Length	0.54	36	0.0004
Dorsal Ray 11 Length	0.55	25	0.0028
Dorsal Ray 12 Length	0.44	2	0.5556
Anal Ray #	0.16	37	0.3344
Anal Area	0.50	37	0.0011
Anal Base Length	0.62	37	2.47E‐05
Mean Anal Fin Ray Length	0.49	37	0.0016
Anal Ray 1 Length	0.16	37	0.3237
Anal Ray 2 Length	0.36	37	0.0245
Anal Ray 3 Length	0.43	37	0.0065
Anal Ray 4 Length	0.47	37	0.0025
Anal Ray 5 Length	0.47	37	0.0028
Anal Ray 6 Length	0.43	37	0.0060
Anal Ray 7 Length	0.44	37	0.0054
Anal Ray 8 Length	0.46	37	0.0030
Anal Ray 9 Length	0.46	37	0.0033
Anal Ray 10 Length	0.44	32	0.0084
Anal Ray 11 Length	0.37	15	0.1425
Caudal Ray #	0.05	37	0.7754
Caudal Area	0.65	37	8.68E‐06
Caudal Base Length	0.84	37	1.94E‐11
Mean Caudal Fin Ray Length	0.84	37	2.31E‐11
Median Caudal Ray Length	0.84	37	1.98E‐11
Caudal Dorsal 1 Ray Length	0.80	37	1.40E‐09
Caudal Dorsal 2 Ray Length	0.85	37	1.24E‐11
Caudal Dorsal 3 Ray Length	0.82	37	1.24E‐10
Caudal Dorsal 4 Ray Length	0.78	37	3.80E‐09
Caudal Dorsal 5 Ray Length	0.81	37	6.00E‐10
Caudal Dorsal 6 Ray Length	0.71	37	3.74E‐07
Caudal Dorsal 7 Ray Length	0.83	20	2.14E‐06
Caudal Ventral 1 Ray Length	0.80	37	1.49E‐09
Caudal Ventral 2 Ray Length	0.84	37	2.09E‐11
Caudal Ventral 3 Ray Length	0.84	37	1.70E‐11
Caudal Ventral 4 Ray Length	0.81	37	4.69E‐10
Caudal Ventral 5 Ray Length	0.74	37	5.41E‐08
Caudal Ventral 6 Ray Length	0.61	37	3.89E‐05
Caudal Ventral 7 Ray Length	−0.64	2	0.3646
Pelvic Ray #	0.18	36	0.2709
Pelvic Area	0.48	37	0.0018
Pelvic Base Length	0.33	37	0.0418
Mean Pelvic Fin Ray Length	0.48	37	0.0020
Pelvic Ray 1 Length	0.36	37	0.0241
Pelvic Ray 2 Length	0.50	37	0.0011
Pelvic Ray 3 Length	0.47	37	0.0023
Pelvic Ray 4 Length	0.42	37	0.0083
Pelvic Ray 5 Length	0.38	37	0.0171
Pelvic Ray 6 Length	0.27	32	0.1185
Pectoral Ray #	0.11	35	0.5263
Pectoral Area	0.58	35	0.0001
Pectoral Base Length	0.18	35	0.2978
Mean Pectoral Fin Ray Length	0.73	35	2.22E‐07
Pectoral Ray 1 Length	0.25	35	0.1306
Pectoral Ray 2 Length	0.46	35	0.0044
Pectoral Ray 3 Length	0.54	35	0.0006
Pectoral Ray 4 Length	0.55	35	0.0004
Pectoral Ray 5 Length	0.65	35	1.40E‐05
Pectoral Ray 6 Length	0.70	35	1.28E‐06
Pectoral Ray 7 Length	0.62	34	5.04E‐05
Pectoral Ray 8 Length	0.52	33	0.0012
Pectoral Ray 9 Length	0.52	31	0.0017
Pectoral Ray 10 Length	0.42	22	0.0434
Pectoral Ray 11 Length	0.56	11	0.0450
Pectoral Ray 12 Length	0.86	3	0.0648

**FIGURE 3:**
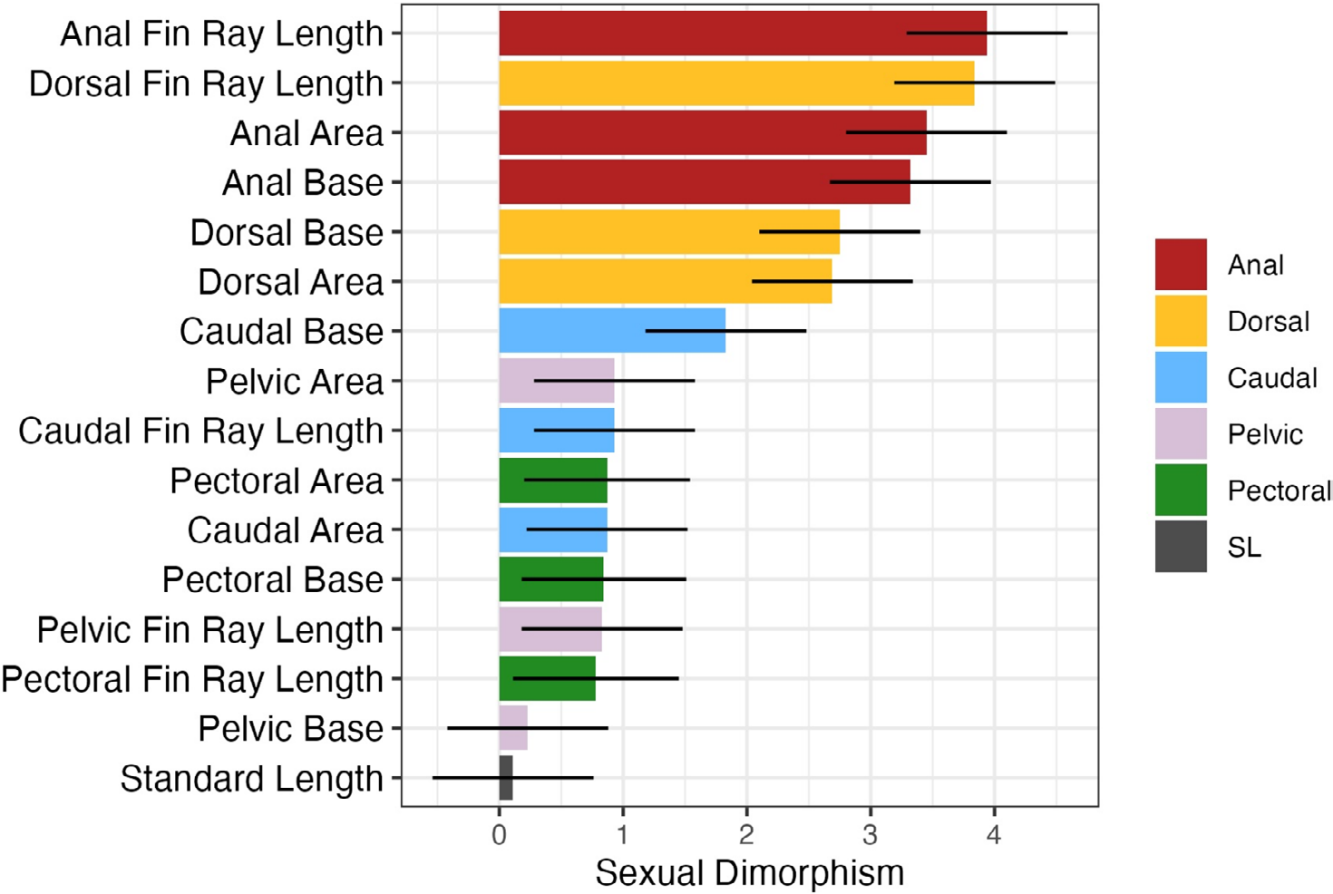
Sexual dimorphism for anal, dorsal, caudal, pelvic, and pectoral fins and standard length. Effect sizes and 95% confidence intervals are shown.

**FIGURE 4:**
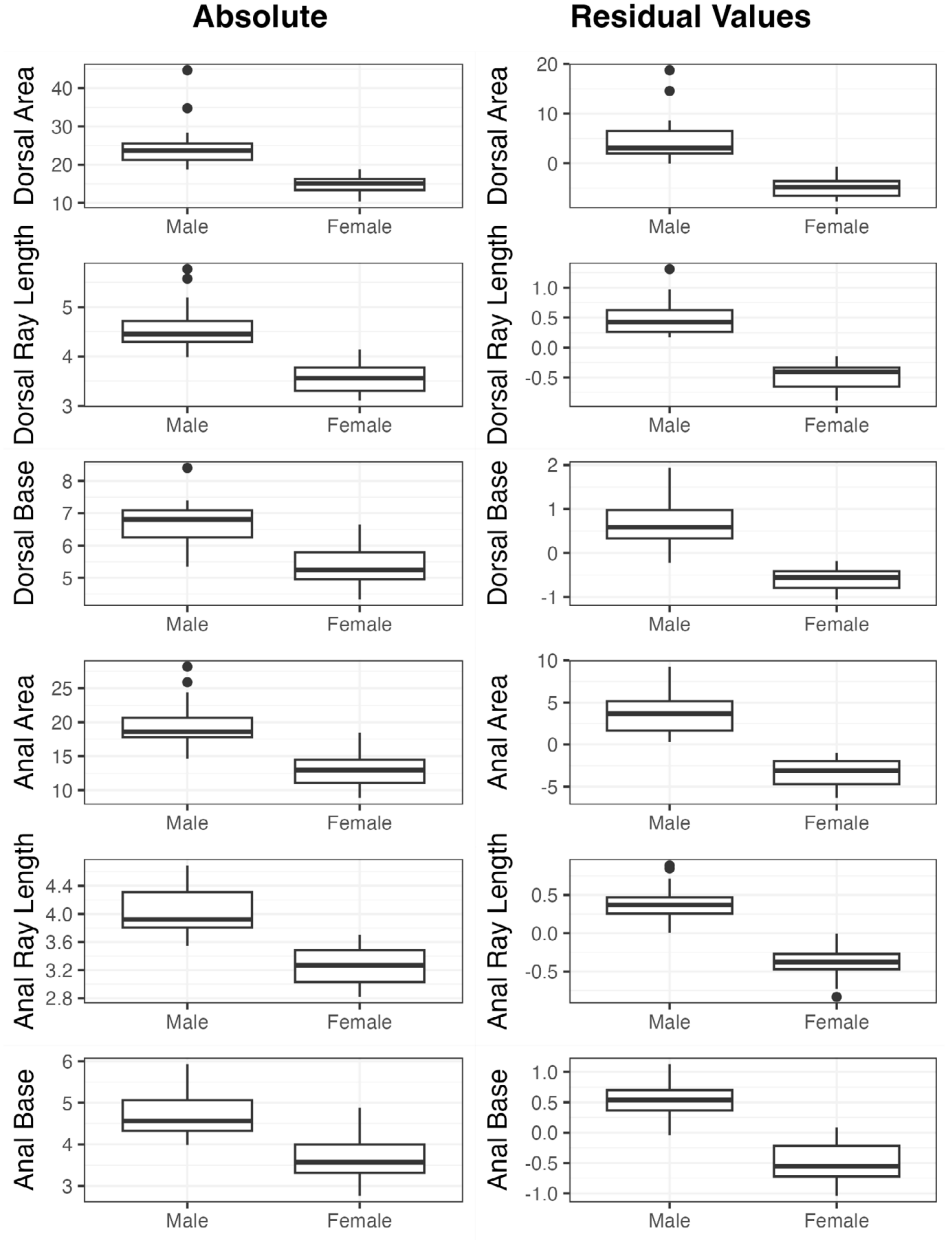
Raw and residual values for dorsal and anal fin traits as a function of sex. Residual values are from a linear regression of raw trait values on standard length. Areas are in units of mm^2^. Lengths are in units of mm.

**FIGURE 5:**
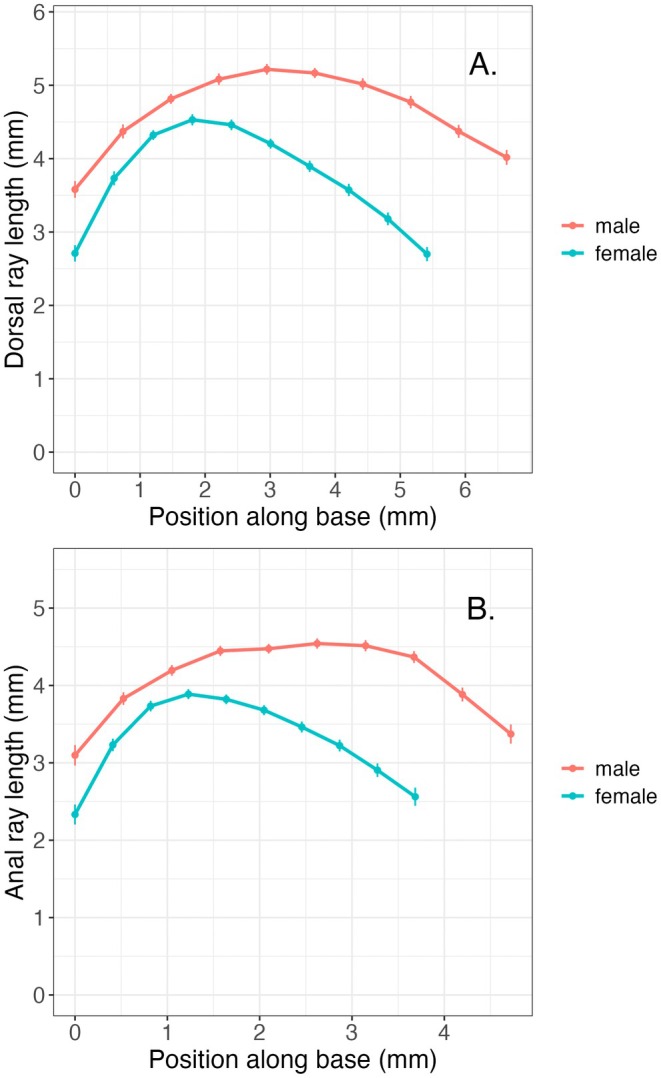
Least‐square means ± SE of ray lengths versus relative position along the fin base relative to the insertion point (0) and the sex‐specific average fin base length for (A) dorsal and (B) anal fins. The figures indicate the average size and shape of dorsal and anal fins in males and females.

The overarching goal of this study was to determine which fins had the highest levels of sexual dimorphism. Anal and dorsal fins were much more sexually dimorphic than caudal, pelvic, or pectoral fins (Tables 3 and 4, Figures 3, 4, 5). On average, dorsal and anal fin areas were 68% and 55% larger, respectively, in males than in females. Average dorsal and anal fin ray lengths were particularly dimorphic (3.84 and 3.94, respectively). On average, anal and dorsal fin traits were 3× more dimorphic than caudal, pectoral, or pelvic fins. Figure 4 shows the raw and size‐adjusted values for the dorsal and anal fin traits. Fin area, fin ray length, and fin base length all showed signs of high sexual dimorphism, suggesting that sexual dimorphism in both base length and ray length contributes to the strong signature of sexual dimorphism in fin area.

**TABLE 3:** Analyses of variance as a function of sex and population for size corrected traits.

Trait	Model	Sex	Population	Sex dimorph (95% CL)
Dorsal Area	***35.27***	***70.42***	0.01	2.69 (2.04, 3.34)
	***3.30E‐09***	***5.39E‐10***	0.9083	
Dorsal Base	***36.99***	***73.74***	0.07	2.75 (2.1, 3.4)
	***1.86E‐09***	***3.08E‐10***	0.7862	
Dorsal Fin Ray Length	***71.98***	***143.58***	0.87	3.84 (3.19, 4.49)
	***2.63E‐13***	***4.01E‐14***	0.3583	
Anal Base	***53.73***	***107.25***	0.03	3.32 (2.67, 3.97)
	***1.56E‐11***	***2.42E‐12***	0.8605	
Anal Area	***57.92***	***115.74***	0.00	3.45 (2.8, 4.1)
	***5.60E‐12***	***8.51E‐13***	0.9721	
Anal Fin Ray Length	***76.81***	***150.86***	3.95	3.94 (3.29, 4.59)
	***1.03E‐13***	***1.95E‐14***	0.0546	
Caudal Base	***16.67***	***32.44***	0.63	1.83 (1.18, 2.48)
	***7.51E‐06***	***1.77E‐06***	0.4309	
Caudal Area	*3.88*	*7.4*	0.29	0.87 (0.22, 1.52)
	*0.0297*	*0.0100*	0.5966	
Caudal Fin Ray Length	*4.54*	***8.46***	0.75	0.93 (0.28, 1.58)
	*0.0174*	***0.0062***	0.3910	
Pelvic Base	0.73	0.53	0.96	0.23 (−0.42, 0.88)
	0.4906	0.4717	0.3337	
Pelvic Area	*4.32*	***8.42***	0.15	0.93 (0.28, 1.58)
	*0.0208*	***0.0063***	0.6993	
Pelvic Fin Ray Length	*4.22*	*6.68*	1.94	0.83 (0.18, 1.48)
	*0.0226*	*0.0139*	0.1720	
Pectoral Base	*3.44*	*6.59*	0.37	0.84 (0.18, 1.51)
	*0.0437*	*0.0148*	0.5459	
Pectoral Area	*3.58*	*7.03*	0.19	0.87 (0.2, 1.54)
	*0.0388*	*0.0121*	0.6658	
Pectoral Fin Ray Length	*3.14*	*5.62*	0.56	0.78 (0.11, 1.45)
	*0.0558*	*0.0235*	0.4610	

*Note:* All trait values are residuals from a linear regression on standard length. *F*‐values are listed on top and *p*‐values below. Values in plain italic have significant unadjusted *p*‐values. Values in bold italic remain statistically significant after a sequential Bonferroni (“holm”) adjustment. The levels of sexual dimorphism are calculated as the effect size due to sex plus their 95% confidence limits. Denominator DF are 36 for all models except for pectoral fin traits where they are 34.

**TABLE 4:** Analyses of variance on individual size‐adjusted fin ray lengths.

Trait	Model *F*	Sex *F*	Source *F*	Sex dimorph	Denom DF
Dorsal R 1	***14.89***	***29.76***	0.00	1.75 (1.1, 2.4)	36
Dorsal R 2	***11.33***	***22.63***	0.10	1.52 (0.87, 2.17)	36
Dorsal R 3	***13.44***	***26.89***	0.01	1.66 (1.01, 2.31)	36
Dorsal R 4	***12.78***	***25.55***	0.06	1.62 (0.97, 2.27)	36
Dorsal R 5	***27.33***	***54.6***	0.17	2.37 (1.72, 3.02)	36
Dorsal R 6	***51.76***	***103.4***	0.39	3.26 (2.61, 3.91)	36
Dorsal R 7	***52.72***	***105.24***	0.53	3.29 (2.64, 3.94)	36
Dorsal R 8	***50.35***	***100.58***	0.38	3.21 (2.56, 3.86)	36
Dorsal R 9	***47.3***	***94.37***	0.55	3.11 (2.46, 3.76)	36
Dorsal R 10	***47.47***	***94.21***	1.95	3.16 (2.5, 3.82)	35
Anal R 1	***8.87***	***17.36***	0.52	1.34 (0.69, 1.99)	36
Anal R 2	***14.29***	***28.12***	0.67	1.7 (1.05, 2.35)	36
Anal R 3	***12.09***	***23.51***	0.89	1.55 (0.9, 2.2)	36
Anal R 4	***21.48***	***40.69***	2.80	2.04 (1.39, 2.69)	36
Anal R 5	***30.17***	***59.31***	1.49	2.47 (1.82, 3.12)	36
Anal R 6	***44.74***	***89.05***	0.81	3.02 (2.37, 3.67)	36
Anal R 7	***54.35***	***108.63***	0.29	3.34 (2.69, 3.99)	36
Anal R 8	***58.65***	***116.85***	0.91	3.46 (2.81, 4.11)	36
Anal R 9	***30.81***	***61.44***	0.41	2.51 (1.86, 3.16)	36
Anal R 10	***13.64***	***26.19***	2.81	1.77 (1.06, 2.48)	31
Median Caudal R	1.86	3.01	0.80	0.56 (−0.09, 1.21)	36
Caudal Dorsal R 1	2.11	3.51	0.79	0.6 (−0.05, 1.25)	36
Caudal Dorsal R 2	1.18	1.9	0.51	0.44 (−0.21, 1.09)	36
Caudal Dorsal R 3	2.22	3.95	0.58	0.64 (−0.01, 1.29)	36
Caudal Dorsal R 4	1.27	2.46	0.10	0.5 (−0.15, 1.15)	36
Caudal Dorsal R 5	1.48	2.43	0.59	0.5 (−0.15, 1.15)	36
Caudal Dorsal R 6	0.82	1.15	0.53	0.34 (−0.31, 0.99)	36
Caudal Ventral R 1	2.51	*4.82*	0.25	0.7 (0.05, 1.35)	36
Caudal Ventral R 2	1.24	2.29	0.23	0.49 (−0.16, 1.14)	36
Caudal Ventral R 3	1.4	2.53	0.31	0.51 (−0.14, 1.16)	36
Caudal Ventral R 4	2.32	*4.44*	0.25	0.68 (0.03, 1.33)	36
Caudal Ventral R 5	6.23	***12.15***	0.43	1.12 (0.47, 1.77)	36
Caudal Ventral R 6	*6.74*	***12.22***	1.47	1.12 (0.47, 1.77)	36
Pelvic R 1	2.93	1.92	4.09	0.44 (−0.21, 1.09)	36
Pelvic R 2	2.41	2.19	2.76	0.47 (−0.18, 1.12)	36
Pelvic R 3	**8.26**	***15.65***	1.08	1.27 (0.62, 1.92)	36
Pelvic R 4	5.17	*9.8*	0.67	1 (0.35, 1.65)	36
Pelvic R 5	2.16	*4.3*	0.03	0.66 (0.01, 1.31)	36
Pelvic R 6	0.41	0.6	0.26	0.27 (−0.43, 0.97)	31
Pectoral R 1	0.03	0	0.07	−0.02 (−0.69, 0.65)	34
Pectoral R 2	0.89	0.82	0.91	0.3 (−0.37, 0.97)	34
Pectoral R 3	2.74	*4.13*	1.20	0.67 (0, 1.34)	34
Pectoral R 4	2.19	3.75	0.54	0.64 (−0.03, 1.31)	34
Pectoral R 5	**7.93**	***14.85***	0.80	1.27 (0.6, 1.94)	34
Pectoral R 6	4.19	*7.96*	0.31	0.93 (0.26, 1.6)	34
Pectoral R 7	0.9	1.69	0.11	0.43 (−0.25, 1.11)	33
Pectoral R 8	0.91	1.8	0.02	0.45 (−0.23, 1.14)	32
Pectoral R 9	1.18	2.33	0.00	0.53 (−0.18, 1.25)	30

*Note:* Adjusted fin ray lengths were residuals from a regression on standard length. Values in *plain* italic have unadjusted *p*‐values < 0.05. *F*‐values in bold italic remain statistically significant after a sequential Bonferroni (“holm”) correction.

Anal and dorsal fins were particularly dimorphic for the posterior fin rays. Table 4 shows the levels of sexual dimorphism for each fin ray. Posterior dorsal and anal fin ray lengths had sexual dimorphism levels that were ~2× higher than those for some anterior fin rays. Figure 5 shows the least square means for each fin ray versus the relative position of each ray given the fin base length as a function of sex. The enlarged posterior dorsal and anal fin rays positioned further along the body axis make the fin area larger, particularly on the posterior portion of the fin.

Caudal, pelvic, and pectoral fins also showed moderate levels of sexual dimorphism, with males having larger fin areas and longer fin rays than females. Males also had larger caudal and pectoral fin base lengths than did females. These effects were modest.

This study also asked whether the levels of variability vary as a function of sex, particularly for traits with high levels of sexual dimorphism. Size‐corrected dorsal fin area and base length were ~2× more variable in males than in females (Table 5, Figure 4). Average pelvic ray length and the lengths of pelvic rays 1–3 were ~2× more variable in females than males despite being smaller in magnitude in females. However, a sequential Bonferroni correction rendered these patterns statistically non‐significant. Overall, the levels of variability were remarkably similar between males and females across traits. Coefficients of variation ranged from ~0.05 for average fin ray lengths to ~0.20 for some fin areas. In general, the levels of variability were not consistently higher for traits with high levels of sexual dimorphism.

**TABLE 5:** Mean values, coefficients of variation, and *p*‐values from Levene tests for tests of differences in variance between males and females for raw values and residual trait values.

Trait	Mean (♂)	Mean (♀)	Raw CV (♂)	Raw CV (♀)	Resid CV (♂)	Resid CV (♀)	Levene raw	Levene resid values
**Ray numbers**								
Dorsal	10.49	10.84	0.069	0.064	0.067	0.055	0.538	0.321
Anal	10.05	10.38	0.073	0.093	0.072	0.085	0.344	0.749
Caudal	13.47	13.80	0.045	0.050	0.045	0.049	0.689	0.642
Pelvic	5.78	5.85	0.061	0.079	0.060	0.078	0.738	0.552
Pectoral	9.79	9.42	0.134	0.134	0.134	0.132	0.839	0.883
**Fin area**								
*Dorsal*	*24.71*	*14.69*	*0.249*	*0.159*	*0.190*	*0.100*	*0.056*	*0.010*
Anal	19.74	12.72	0.173	0.205	0.120	0.118	0.382	0.094
Caudal	44.87	38.97	0.153	0.258	0.133	0.154	0.262	0.897
Pelvic	7.63	6.09	0.237	0.313	0.156	0.297	0.923	0.477
Pectoral	17.57	14.62	0.263	0.246	0.206	0.191	0.427	0.304
**Base length**								
*Dorsal*	*6.65*	*5.40*	*0.111*	*0.123*	*0.085*	*0.047*	*0.774*	*0.013*
Anal	4.73	3.67	0.120	0.153	0.065	0.083	0.923	0.795
Caudal	4.40	4.05	0.097	0.103	0.038	0.046	0.620	0.374
Pelvic	1.32	1.27	0.178	0.204	0.162	0.197	0.566	0.524
Pectoral	2.02	1.77	0.172	0.131	0.167	0.130	0.127	0.119
**Mean ray length**								
Dorsal	4.58	3.59	0.106	0.090	0.063	0.052	0.291	0.233
Anal	4.05	3.25	0.093	0.081	0.054	0.055	0.113	0.515
Caudal	5.51	5.25	0.086	0.100	0.041	0.053	0.930	0.242
*Pelvic*	*3.20*	*2.87*	*0.107*	*0.193*	*0.060*	*0.181*	*0.010*	*0.007*
Pectoral	3.83	3.57	0.138	0.118	0.095	0.068	0.190	0.131
**Dorsal**								
Ray 1	3.59	2.71	0.179	0.148	0.166	0.134	0.252	0.239
Ray 2	4.38	3.73	0.131	0.121	0.105	0.097	0.930	0.720
Ray 3	4.82	4.32	0.085	0.093	0.053	0.074	0.809	0.551
Ray 4	5.09	4.52	0.096	0.091	0.072	0.067	0.874	0.830
Ray 5	5.23	4.45	0.105	0.089	0.067	0.059	0.562	0.626
Ray 6	5.18	4.20	0.100	0.097	0.059	0.063	0.742	0.700
Ray 7	5.03	3.89	0.112	0.113	0.076	0.073	0.339	0.065
Ray 8	4.78	3.57	0.117	0.132	0.086	0.091	0.435	0.081
Ray 9	4.38	3.17	0.139	0.159	0.094	0.108	0.784	0.268
Ray 10	4.02	2.70	0.181	0.226	0.107	0.152	0.942	0.649
Ray 11	3.36	2.27	0.221	0.217	0.077	0.157	0.251	0.889
**Anal**								
Ray 1	3.10	2.33	0.181	0.257	0.169	0.257	0.822	0.736
Ray 2	3.83	3.23	0.113	0.108	0.078	0.107	0.238	0.968
Ray 3	4.20	3.73	0.089	0.085	0.064	0.081	0.266	0.837
Ray 4	4.45	3.88	0.084	0.084	0.055	0.076	0.250	0.391
Ray 5	4.48	3.82	0.086	0.079	0.063	0.064	0.571	0.821
Ray 6	4.55	3.68	0.091	0.090	0.068	0.069	0.497	0.283
Ray 7	4.52	3.46	0.101	0.114	0.076	0.080	0.576	0.077
Ray 8	4.37	3.22	0.119	0.132	0.077	0.097	0.471	0.461
Ray 9	3.89	2.90	0.132	0.168	0.105	0.126	0.744	0.949
Ray 10	3.36	2.57	0.183	0.222	0.168	0.157	0.976	0.241
Ray 11	2.96	2.65	0.146	0.165	0.129	0.117	0.751	0.995
**Caudal**								
D Ray 7	4.40	4.40	0.147	0.156	0.096	0.080	0.790	0.728
D Ray 6	5.19	5.01	0.112	0.150	0.079	0.103	0.851	0.650
D Ray 5	5.64	5.44	0.097	0.126	0.050	0.077	0.624	0.085
D Ray 4	5.76	5.55	0.096	0.118	0.056	0.075	0.864	0.056
D Ray 3	5.72	5.52	0.089	0.098	0.045	0.057	0.929	0.159
D Ray 2	5.63	5.50	0.086	0.092	0.044	0.050	0.846	0.381
D Ray 1	5.58	5.39	0.089	0.097	0.048	0.061	0.974	0.516
Med Ray	5.60	5.44	0.083	0.093	0.043	0.050	0.930	0.330
V Ray 1	5.62	5.39	0.075	0.109	0.040	0.066	0.632	0.551
V Ray 2	5.62	5.48	0.076	0.093	0.046	0.043	0.695	0.534
V Ray 3	5.65	5.50	0.082	0.092	0.046	0.046	0.978	0.987
V Ray 4	5.67	5.47	0.083	0.091	0.049	0.050	0.893	0.906
V Ray 5	5.49	5.11	0.095	0.102	0.055	0.066	0.927	0.740
V Ray 6	4.91	4.36	0.108	0.168	0.074	0.132	0.134	0.287
**Pelvic**								
*Ray 1*	*2.61*	*2.41*	*0.136*	*0.248*	*0.109*	*0.240*	*0.079*	*0.007*
*Ray 2*	*3.30*	*3.09*	*0.126*	*0.195*	*0.079*	*0.182*	*0.047*	*0.003*
*Ray 3*	*3.88*	*3.37*	*0.087*	*0.167*	*0.055*	*0.152*	*0.002*	*0.001*
Ray 4	3.78	3.21	0.140	0.219	0.090	0.212	0.150	0.093
Ray 5	3.12	2.73	0.173	0.248	0.137	0.241	0.666	0.705
Ray 6	2.44	2.29	0.191	0.292	0.184	0.280	0.368	0.770
**Pectoral**								
Ray 1	2.51	2.51	0.390	0.350	0.382	0.333	0.432	0.473
Ray 2	3.63	3.42	0.200	0.218	0.173	0.197	0.545	0.440
Ray 3	4.33	3.95	0.157	0.162	0.136	0.127	0.987	0.795
Ray 4	4.67	4.34	0.117	0.148	0.106	0.109	0.478	0.753
Ray 5	4.91	4.41	0.097	0.140	0.069	0.097	0.615	0.951
Ray 6	4.73	4.34	0.119	0.143	0.086	0.091	0.790	0.613
Ray 7	4.35	4.09	0.194	0.161	0.161	0.107	0.247	0.090
Ray 8	3.81	3.49	0.236	0.222	0.205	0.179	0.367	0.342
Ray 9	3.18	2.80	0.346	0.291	0.277	0.249	0.050	0.117
Ray 10	2.48	2.49	0.399	0.310	0.347	0.291	0.224	0.207

*Note:* Values in plain italic were statistically significant in the initial Levene test. None of the results were significant after a Bonferroni sequential correction. For caudal rays, D = dorsal, med = median, V = ventral.

**FIGURE 6:**
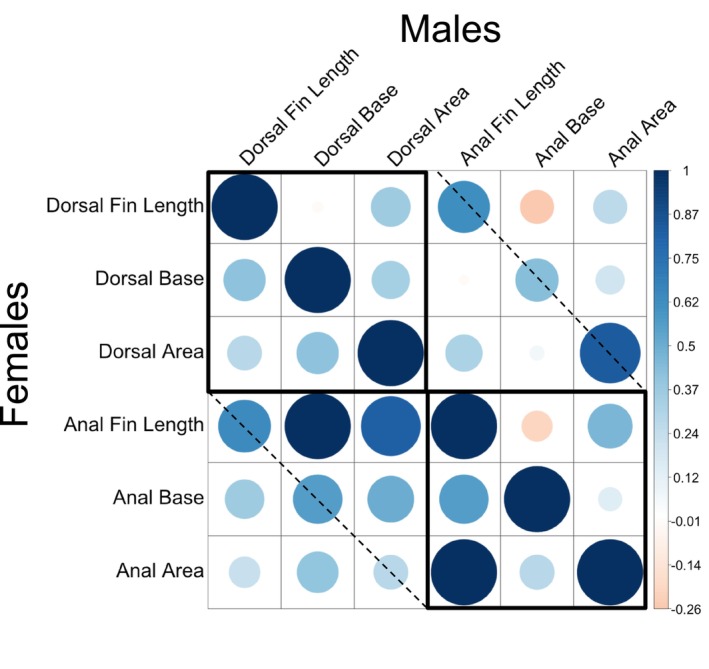
Correlations between fin ray length, fin base length, and fin area in dorsal and anal fins for males (top) and females (bottom). Bold squares show correlations between elements within a fin type. The dashed line shows the correlation between the same fin trait (i.e., fin ray lengths) between the dorsal and anal fins.

Size‐corrected anal fin and dorsal fin traits were strongly correlated with one another in both sexes. Size‐adjusted dorsal and anal fin areas were strongly correlated in males (Table 6, Figure 6, *R*_17_ = 0.84, 95% CL: 0.94, 0.62) and moderately correlated in females (Table 6). There were positive correlations among most of the fin areas for both sexes, but only the correlation between size‐corrected dorsal and anal fin area remained statistically significant after a sequential Bonferroni correction in males. Furthermore, the 95% confidence intervals for the correlation between dorsal and anal fins excluded the other correlation coefficients between other fin area traits. The correlation between dorsal and anal fin area was also high in females (and was the highest correlation among fin areas), but it was not statistically significant after a sequential Bonferroni correction (Table 6, Figure 6).

**TABLE 6:** Pearson correlation coefficients between size‐corrected dorsal, anal, caudal, pelvic, and pectoral fin traits (A: Fin Area, B: Average Fin Ray Length, C: Base Length).

	Dorsal	Anal	Caudal	Pelvic	Pectoral
**(A: Fin Area)**					
Dorsal		***0.84***	*0.59*	*0.49*	0.37
Anal	*0.51*		0.51	*0.47*	0.34
Caudal	0.16	0.33		0.42	0.04
Pelvic	0.22	*0.54*	−0.04		*0.51*
Pectoral	0.12	0.28	0.2	0.29	
**(B: Ray Length)**					
Dorsal		***0.62***	0.29	0.43	0.04
Anal	***0.63***		0.37	0.4	0.21
Caudal	*0.47*	*0.45*		0.06	0.05
Pelvic	*0.47*	*0.59*	0.35		0.06
Pectoral	0.41	0.01	0.1	0.41	
**(C: Base Length)**					
Dorsal		0.43	*0.48*	0.1	−0.07
Anal	***0.82***		0.15	0	−0.02
Caudal	−0.1	−0.33		−0.07	0.15
Pelvic	0.34	*0.45*	−0.15		−0.34
Pectoral	0.34	0.22	0.09	−0.08	

*Note:* Values above the diagonal are correlations across males. Values below the diagonal are correlations across females. Values in bold italic remain statistically significant after a sequential Bonferroni (“holm”) correction. Values in plain italic have unadjusted *p*‐values < 0.05.

Similar patterns were seen for fin ray lengths and base lengths (Table 6, Figure 6). Dorsal and anal fin ray lengths were strongly correlated with one another in both males and females, and both remained statistically significant after a sequential Bonferroni correction. For both males and females, the correlations between dorsal and anal fin ray lengths were higher than the correlations among fin ray lengths for other combinations of fins. Females also had a high correlation between dorsal and anal fin base lengths (*R*_18_ = 0.84), which was nearly 2× higher than any of the other correlations for base lengths. Among males, there were positive correlations between dorsal and caudal fin base lengths, but they did not remain statistically significant after a sequential Bonferroni correction. While dorsal fin traits (i.e., fin ray length, fin area) were correlated with anal fin traits, there were not strong correlations between fin ray length and fin base length, particularly in males (Figure 6).

## 4 Discussion

In this study, we sought to determine the degree of sexual dimorphism in fin morphology in the bluefin killifish, *Lucania goodei*. The results paint a picture of high sexual dimorphism in dorsal and anal fins relative to the other fins (pelvic, pectoral, and caudal fins). The greater fin area in both dorsal and anal fins was due to increases in both fin base length and fin ray length. The dimorphism was particularly pronounced in the posterior fin rays for both the dorsal and anal fins. The number of fin rays was not dimorphic, a finding consistent with the use of fin rays as diagnostic characteristics of species (Page and Burr 2011). Our finding of high sexual dimorphism in dorsal and anal fins is in keeping with the findings of Davis et al. (2025). Davis et al. (2025) measured sexual dimorphism in dorsal, anal, and caudal fins across 20 species of Fundulidae, including bluefin killifish, and found high levels of dimorphism in dorsal and anal fins and lower levels in caudal fins. While the concordance is not surprising, it is reassuring. The present study builds on prior work by using fresh specimens, a larger sample size, and by incorporating measurements of the paired fins (i.e., pectoral and pelvic fins). The larger sample size also enabled investigation of trait correlations within each sex.

One of the major patterns to emerge is that dorsal and anal fins have much higher levels of sexual dimorphism than those found in caudal, pectoral, and pelvic fins, suggesting that anal and dorsal fins are under strong sexual selection. Similar results have been found in other groups. Mainero et al. (2023) performed a similar study in the Arabian killifish, *Aphaniops stoliczkanus*, from the family Aphaniidae, and found a similar pattern with high sexual dimorphism in dorsal and anal fins and lower levels in pectoral and pelvic fins. In a beach‐spawning capelin, *Mallotus villosus*, sexual dimorphism was found to be particularly high in the anal fins, but other fins were less so (Orbach et al. 2019). Englmaier et al. (2022) found apparent high levels of sexual dimorphism in dorsal and anal fins and lower levels in other fins. Elevated dimorphism in dorsal and anal fins has been documented across a range of lineages, including gars, blennies, minnows, cichlids, and salmonids (Chervinski 1965; McCart 1965; Ostrand et al. 2001; McGrath and Hilton 2012; Englmaier et al. 2022). This strikingly consistent pattern suggests that several distantly related species face similar selection pressures.

The question of why dorsal and anal fins frequently show such high levels of sexual dimorphism is not fully resolved. Dorsal and anal fins are often involved in signaling between males and females (Thompson and Sturm 1965; Fuller 2001; McGhee et al. 2007; Johnson and Fuller 2015; Zhou et al. 2015; Mitchem et al. 2018). However, a more compelling answer may be that many of these species pair spawn. In many species, males use their dorsal and anal fins to clasp females and guide gamete release (Newman 1907; Foster 1967; Arndt 1971; Able and Hata 1984). The anal fin, in particular, may play a large role in controlling the direction of the flow of sperm and eggs, and preventing sperm of other males from reaching the eggs (Barbas and Gilg 2018). Experimental work supports this idea: in medaka, shortening the anal fin reduces both fertilization success and male attractiveness to females (Koseki et al. 2000; Fujimoto et al. 2014). Similar results have been found in bluefin killifish, where altering the male anal fin has strong effects on fertilization success and moderate effects on male/male competition and female mating preferences (Smelko and Fuller 2025).

More challenging is the question of why the caudal, pectoral, and pelvic fins also exhibit moderate dimorphism in some species. In some taxa, paired fins have clearly evolved mating‐related functions. In sticklebacks, for instance, sexual dimorphism in pectoral fins is associated with male parental care (Bakker and Mundwiler 1999), and experimental reductions in pectoral fin size alter fanning behavior and possibly other life‐history traits (Künzler and Bakker 2000). Pelvic fins are often dramatically dimorphic. In the cichlid *Cyathopharynx fucifer*, males use elongated pelvic fins in courtship displays (Karino 1997). In one species of ricefish, females brood embryos below their abdomen and have evolved modified pelvic fins to protect the offspring (Flury et al. 2022). However, in many other species, these fins lack an obvious reproductive function, and the pattern of strong dimorphism in dorsal and anal fins, with weaker or absent dimorphism in other fins, persists.

Why do fins differ in their degree of sexual dimorphism? One possibility is that overall fin growth is weakly correlated across fin types, such that strong selection on dorsal or anal fins produces indirect changes in others. Alternatively, fins may vary in the relative intensity of natural versus sexual selection, leading to differing dimorphism levels. Resolving these possibilities will require direct measurements of selection acting on fin morphology.

This study also found strong correlations between dorsal and anal fin traits in both males and females, suggesting a combination of pleiotropy, shared developmental pathways, and/or correlated selection. Comparative studies have explored the modularity of fin traits, asking whether certain fins evolve in concert while others evolve more independently. Several authors have identified a distinct “dorsal‐anal fin module,” in which traits of the dorsal and anal fins are more tightly integrated with one another than with other fins. For example, studies of fin positioning have shown that dorsal and anal fins form a module that is relatively independent from modules involving the pectoral and pelvic fins (Mabee et al. 2002; Larouche et al. 2015). The dorsal and anal fin module appears to have been present in Actinopterygians for at least 400 MY (Mabee et al. 2002).

Further support for shared developmental control comes from QTL studies in medaka. Kawajiri et al. (2014) crossed two medaka populations (one exhibiting low and the other high sexual dimorphism in dorsal and anal fin size) and found that nearly all QTL for dorsal fin traits co‐localized with QTL for anal fin traits. Although some additional QTL were unique to the anal fin, the overlap strongly supports pleiotropic effects in medaka. Similar genetic studies have not yet been conducted in bluefin killifish. However, developmental studies show that dorsal and anal fins emerge simultaneously during embryogenesis and follow similar developmental trajectories during early life stages (Crawford and Balon 1994a, 1994c, 1994b). Further work is needed to determine the extent to which genetic correlations due to pleiotropy occur in bluefin killifish.

A key question, however, is how sexual dimorphism arises within these shared developmental modules. Several lines of evidence suggest that fin growth is sensitive to sex hormones. In medaka, dorsal and anal fins follow similar developmental patterns early in ontogeny but diverge between sexes at the same time that sex hormone levels begin to differ (Kawajiri et al. 2014, 2015; Zhang et al. 2023). Furthermore, androgen treatments in females can induce male‐specific traits, such as the papillary process typically found on the anal fins in male medaka (Ogino et al. 2014). In guppies, mosquitofish, and platyfish, treating females with androgens causes their anal fin to elongate and form a structure that resembles a gonopodium (Turner 1942; Sangster 1948; Hopper 1949; Angus et al. 2001; Offen et al. 2013). In bluefin killifish, Fuller and Travis (2004) demonstrated that methyltestosterone administration induces male‐like coloration patterns in females. Moreover, the presence of androgen, estrogen, and progesterone receptors in the dorsal and anal fins has been documented in several species (Ngamniyom et al. 2009; Kawajiri et al. 2014; Zhang et al. 2023), including bluefin killifish (Karatgi and Fuller, in preparation). These findings suggest that an ancient developmental pathway is likely present and that alteration of growth patterns with sex hormones possibly allows for sexual dimorphism in these fins.

Finally, this study allowed us to examine how trait variability differs across fin types and between sexes. We found little evidence that traits with high levels of sexual dimorphism were more or less variable than other traits, nor did we find consistent differences in trait variability between males and females. Dorsal fin area and dorsal base length were more variable in males than in females, and pelvic fin ray lengths were more variable in females than in males, but these patterns did not hold up after corrections for multiple tests. Over time, expectations regarding variability in male secondary sexual traits have shifted. Early models of sexual selection, including both “good genes” and Fisherian frameworks, predicted that strong selection on male traits would erode genetic variation (Charlesworth 1987; Falconer 1996). However, later models incorporating condition‐dependent expression suggested greater potential for both genetic and environmental variation in male secondary sexual traits (Rowe and Houle 1996; Houle and Kondrashov 2002). There has also been ongoing debate about whether males or females should be more variable overall. Historically, female hormonal cycles were thought to increase phenotypic variability (Zajitschek et al. 2020). In our data, we found little support for differences in trait variability as a function of sex or degree of sexual dimorphism, though our relatively small sample sizes (~20 individuals per sex) may limit our power to detect such patterns. A simple power analysis indicates that a sample size of 20 per sex would only have high power (0.8) for coefficients of variation that vary 2‐fold. We note that if sexual dimorphism in fin size arises from hormone‐dependent growth, as hypothesized, then male fin traits may be particularly sensitive to fluctuating hormone levels associated with changes in social status or dominance.

In conclusion, we found strikingly high levels of sexual dimorphism in fin area, fin base length, and fin ray lengths of the dorsal and anal fins. Posterior fin rays were particularly dimorphic in both fins. These traits were also strongly correlated across individuals in both sexes: individuals with larger dorsal fins also had larger anal fins, and males with longer dorsal fin rays tended to have longer anal fin rays. Whether these correlations reflect pleiotropy, shared developmental pathways, or strong correlated selection remains unresolved. We also detected moderate levels of sexual dimorphism in the caudal, pelvic, and pectoral fins. Caudal and pelvic fins may play a role in courtship or male–male competition, given that they possess sexual dichromatic coloration. In contrast, the pectoral fins are neither dichromatic nor clearly involved in mating. One explanation is that selection on some fin traits may produce correlated changes in others. Overall, bluefin killifish provide a powerful model for investigating fundamental questions in organismal biology, particularly regarding (1) how genetic and environmental factors influence trait values and their integration, and (2) how natural and sexual selection shape fin morphology, and whether these selective pressures vary across different fin types.

## Author Contributions

**Kasey Brockelsby:** data curation (equal), methodology (equal), visualization (equal), writing – original draft (equal). **Elijah J. Davis:** data curation (equal), investigation (equal), methodology (equal), writing – review and editing (equal). **Valerie Shamsyna:** data curation (equal), methodology (equal). **Olivia A. Roden:** data curation (equal), methodology (equal). **Rebecca C. Fuller:** conceptualization (lead), data curation (equal), formal analysis (equal), project administration (lead), software (lead), supervision (lead), validation (equal), visualization (equal), writing – original draft (equal), writing – review and editing (lead).

## Ethics Statement

All procedures were approved by the Institutional Animal Care and Use Committee at the University of Illinois (protocols #23145 and 22112).

## Conflicts of Interest

The authors declare no conflicts of interest.

## Data Availability

Raw data and R scripts supporting this work can be found at https://doi.org/10.5061/dryad.80gb5mm2x.
